# Supplementary material for: Germline NUP98 Variants in Two Siblings with a Rothmund–Thomson-Like Spectrum: Protein Functional Changes Predicted by Molecular Modeling
Source: Int J Mol Sci. 2023 Feb 16;24(4):4028. doi: 10.3390/ijms24044028 (PMC9965077; doi:10.3390/ijms24044028)
Supplement: Supplementary file 1 [file ijms-24-04028-s001.zip › ijms-2146413-supplementary.pdf]

Supplementary material

Germline *NUP98* Variants in Two Siblings with a Rothmund-Thomson-Like Spectrum: Protein Functional Changes Predicted by Molecular Modeling

Elisa Adele Colombo [1], Michele Valiante [2], Matteo Uggeri [3,4], Alessandro Orro [3], Silvia Majore [2], Paola Grammatico [2], Davide Gentilini [5,6], Palma Finelli [7,8], Cristina Gervasini [1], Pasqualina D'Ursi [3*],and Lidia Larizza[7*]

1 Genetica Medica, Dipartimento di Scienze della Salute, Università degli Studi di Milano, 20142 Milano, Italy
2 Laboratory of Medical Genetics, Department of Experimental Medicine, Sapienza University, San Camillo-Forlanini Hospital, 00152 Roma, Italy
3 Department of Biomedical Sciences National Research Council, Institute for Biomedical Technologies, 20054 Segrate, Italy
4 Department of Pharmacy, Section of Medicinal Chemistry, School of Medical and Pharmaceutical Sciences, University of Genoa, 16132 Genoa, Italy
5 Bioinformatics and Statistical Genomics Unit, IRCCS Istituto Auxologico Italiano, Via Ariosto 13, 20145 Milan, Italy
6 Department of Brain and Behavioral Sciences, University of Pavia, 27100, Pavia, Italy
7 Experimental Research Laboratory of Medical Cytogenetics and Molecular Genetics, IRCCS Istituto Auxologico Italiano, Via Ariosto 13, 20145 Milan, Italy
8 Department of Medical Biotechnology and Translational Medicine, University of Milan, 20133 Milan, Italy

* Correspondence: l.larizza@auxologico.it (L.L.); pasqualina.dursi@itb.cnr.it (P.D.)

Figure S1: *NUP98* gene expression. The *NUP98* cDNA fragment of 281 bp is detected in all tested tissues and lymphoblastoid cell lines (LCLs). LCL C+ positive control, C- negative control, MW molecular weight marker

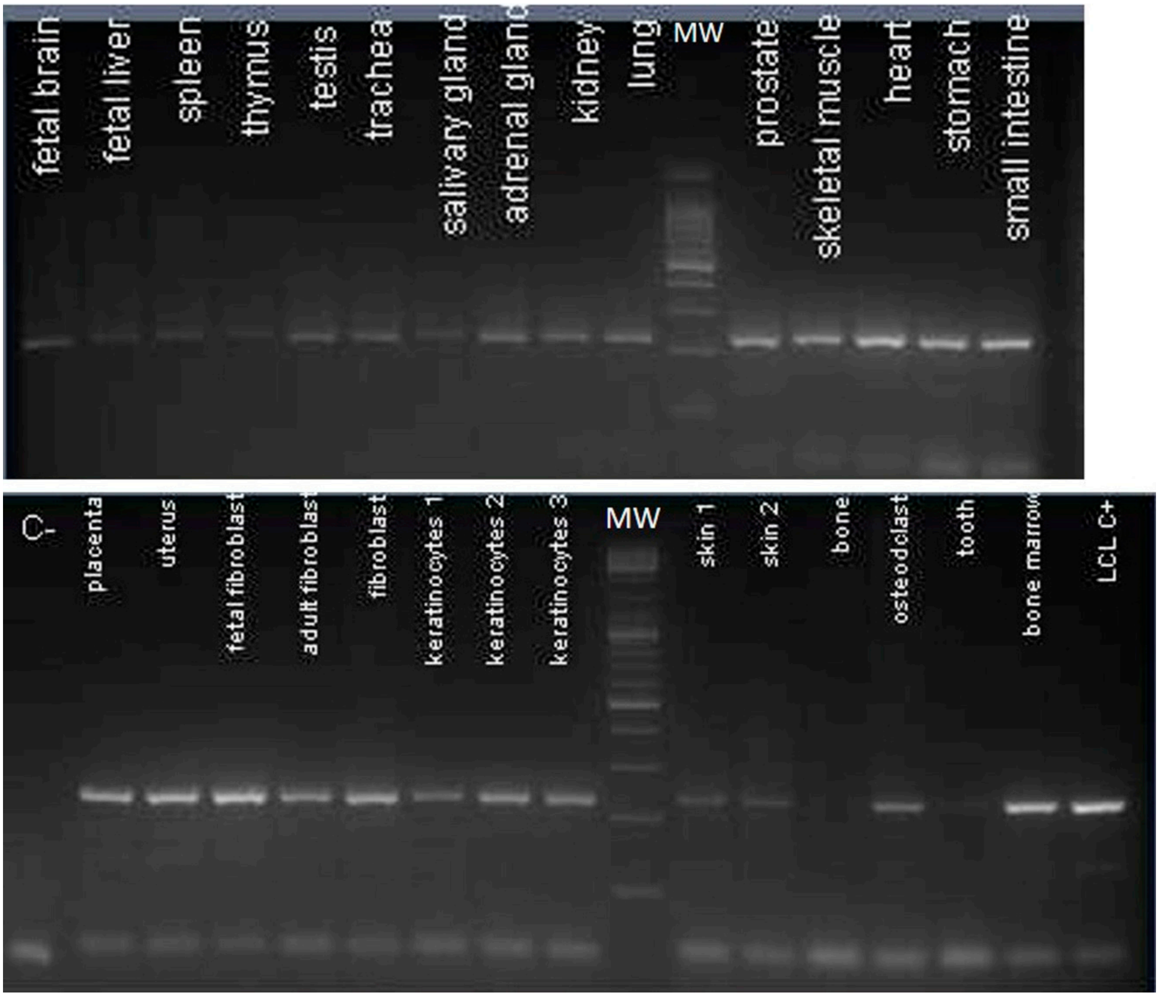

Figure S2: the distances of the F-F pairs. Distance between the centers of mass of the F-F pairs belonging to FG motifs in wild type (blue bars) and G28D (orange bars) NUP98 FG domain.

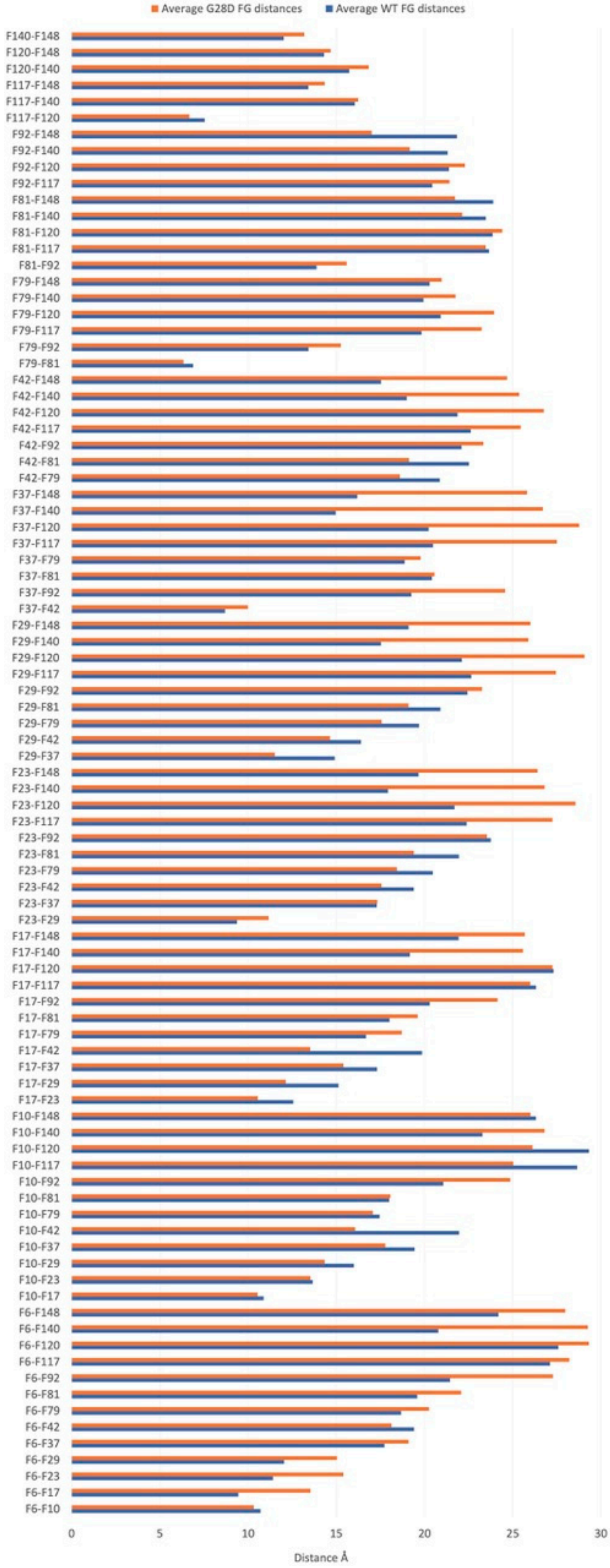

Figure S3: SASA analysis of G28D stable replicas. Results showed that D28 may have to possible orientations: buried (SASA value near or below 0) or exposed (SASA positive value).

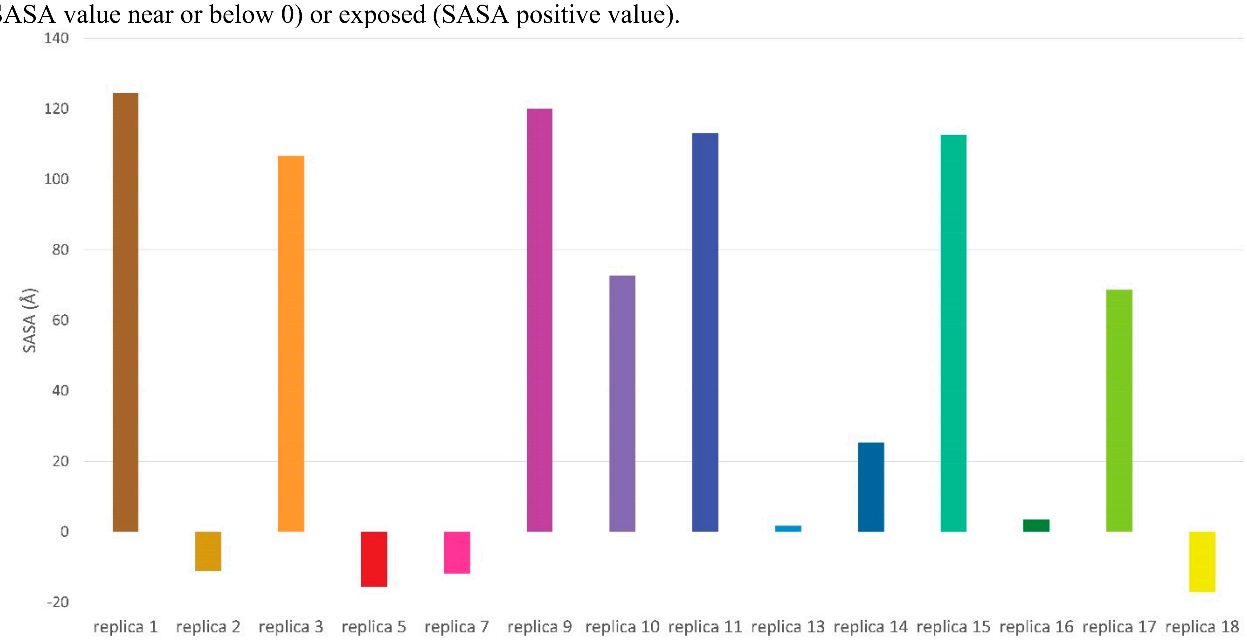

Figure S4: Venn diagram showing common and specific clinical features of NUP98 nucleoporopathy, RTS2 and RTS1.

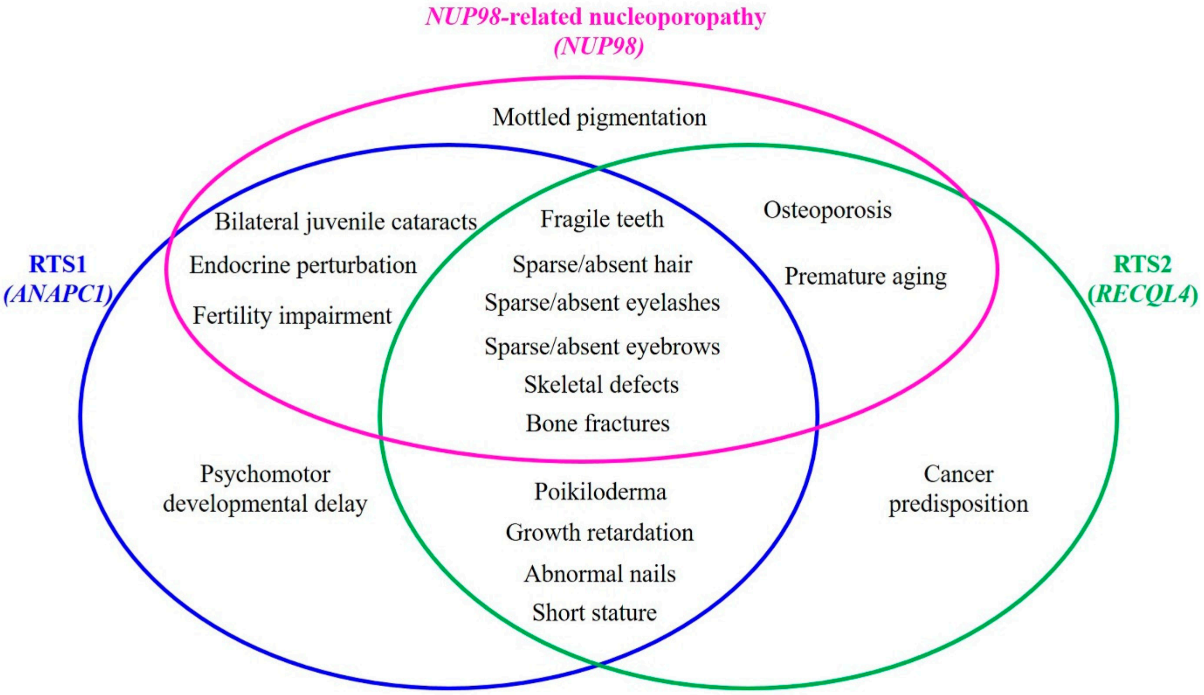

Table S1. Predicted effect at the protein level of *NUP98* variants of the affected siblings.

**Bioinformatic tool**	**Exon 3 variant** c.83G>A (p.Gly28Asp)	**Exon 20 variant** c.2624A>C (p.Glu875Ala)
Polyphen2 [52]	probably damaging	possibly damaging
SIFT [53]	damaging	tolerated
CADD [57]	deleterious	deleterious
P-MUT [58]	disease (85%)	disease (79%)
Panther [59]	probably damaging	possibly damaging
HOPE [60]	might disturb function	might disturb function
Variant effector predictor [61]	moderate impact	moderate impact
Mutation assessor [62]	medium functional impact	medium functional impact
Provean [63]	deleterious	neutral
Mutpred2 [64]	probably damaging	neutral

Table S2. Pprint analysis of the WT and G28D amino acid sequence involved in the RNA interaction.

WT				G28D			
Residue		SVM value	Prediction	Residue		SVM value	Prediction
1	M	-0.55378945	Non-Interacting	1	M	-0.55803765	Non-Interacting
2	F	-0.82641875	Non-Interacting	2	F	-0.87349547	Non-Interacting
3	N	-0.90220625	Non-Interacting	3	N	-0.959148	Non-Interacting
4	K	-0.61748008	Non-Interacting	4	K	-0.6271972	Non-Interacting
5	S	-0.60904646	Non-Interacting	5	S	-0.61693582	Non-Interacting
6	F	-0.3663883	Non-Interacting	6	F	-0.33438624	Non-Interacting
7	G	-0.86719585	Non-Interacting	7	G	-0.91670753	Non-Interacting
8	T	-0.15408726	Interacting	8	T	-0.08689689	Interacting
9	P	-0.38805357	Non-Interacting	9	P	-0.35942944	Non-Interacting
10	F	0.052464119	Interacting	10	F	0.15329547	Interacting
11	G	0.061878645	Interacting	11	G	0.16367349	Interacting
12	G	0.44208841	Interacting	12	G	0.6041482	Interacting
13	G	0.092849556	Interacting	13	G	0.19648674	Interacting
14	T	0.31123103	Interacting	14	T	0.45172504	Interacting
15	G	0.15037959	Interacting	15	G	0.26181989	Interacting
16	G	0.3290125	Interacting	16	G	0.47144538	Interacting
17	F	-0.16312306	Interacting	17	F	-0.07310067	Interacting

18	G	-0.52772285	Non-Interacting	18	G	-0.50382202	Non-Interacting
19	T	-0.54276185	Non-Interacting	19	T	-0.54183776	Non-Interacting
20	T	-0.2921471	Non-Interacting	20	T	-0.25404084	Non-Interacting
21	S	-0.67593245	Non-Interacting	21	S	-0.63787914	Non-Interacting
22	T	-0.53121105	Non-Interacting	22	T	-0.49591728	Non-Interacting
23	F	-0.52116954	Non-Interacting	23	F	-0.51303254	Non-Interacting
24	G	-0.96787691	Non-Interacting	24	G	-1.0327101	Non-Interacting
25	Q	-0.33007003	Non-Interacting	25	Q	-0.35700793	Non-Interacting
26	N	-0.09025613	Interacting	26	N	-0.22664901	Non-Interacting
27	T	0.20832611	Interacting	27	T	-0.09369108	Interacting
28	G	-0.30593648	Non-Interacting	28	D	-0.56705844	Non-Interacting
29	F	0.052686027	Interacting	29	F	-0.09500207	Interacting
30	G	-0.59153605	Non-Interacting	30	G	-0.60180787	Non-Interacting
31	T	0.19650102	Interacting	31	T	0.052826422	Interacting
32	T	-0.0532804	Interacting	32	T	-0.16177192	Interacting
33	S	-0.17722904	Interacting	33	S	-0.17024124	Interacting
34	G	-0.00635643	Interacting	34	G	-0.00468534	Interacting
35	G	-0.19332631	Interacting	35	G	-0.19660448	Interacting
36	A	-0.18253653	Interacting	36	A	-0.17248359	Interacting
37	F	-0.19816187	Interacting	37	F	-0.20855071	Non-Interacting
38	G	-0.84822047	Non-Interacting	38	G	-0.83480983	Non-Interacting
39	T	-0.41467499	Non-Interacting	39	T	-0.41739822	Non-Interacting
40	S	-0.28552534	Non-Interacting	40	S	-0.2767744	Non-Interacting
41	A	-0.32145082	Non-Interacting	41	A	-0.34539552	Non-Interacting
42	F	-0.35849272	Non-Interacting	42	F	-0.34433301	Non-Interacting
43	G	-0.24275524	Non-Interacting	43	G	-0.24665706	Non-Interacting
44	S	-0.38951034	Non-Interacting	44	S	-0.40358287	Non-Interacting
45	S	-0.70910621	Non-Interacting	45	S	-0.69937712	Non-Interacting
46	N	-0.41637418	Non-Interacting	46	N	-0.40777296	Non-Interacting
47	N	0.090789887	Interacting	47	N	0.092923449	Interacting
48	T	0.27514296	Interacting	48	T	0.28303749	Interacting

49	G	0.11327117	Interacting	49	G	0.11327132	Interacting
50	G	0.65111053	Interacting	50	G	0.6350224	Interacting
51	L	-0.02866753	Interacting	51	L	-0.04015428	Interacting
52	F	-0.25792442	Non-Interacting	52	F	-0.25522212	Non-Interacting
53	G	-1.0131399	Non-Interacting	53	G	-0.99196158	Non-Interacting
54	N	0.22322956	Interacting	54	N	0.21665862	Interacting
55	S	-0.21375527	Non-Interacting	55	S	-0.22446886	Non-Interacting
56	Q	-0.09758618	Interacting	56	Q	-0.11153087	Interacting
57	T	0.067543326	Interacting	57	T	0.047492371	Interacting
58	K	0.20446247	Interacting	58	K	0.18253409	Interacting
59	P	0.22663635	Interacting	59	P	0.20503075	Interacting
60	G	-0.11549157	Interacting	60	G	-0.14294754	Interacting
61	G	-0.28683165	Non-Interacting	61	G	-0.31742948	Non-Interacting
62	L	-0.33953353	Non-Interacting	62	L	-0.35158283	Non-Interacting
63	F	-0.73593201	Non-Interacting	63	F	-0.7412335	Non-Interacting
64	G	-0.74425387	Non-Interacting	64	G	-0.73547431	Non-Interacting
65	T	-0.78060069	Non-Interacting	65	T	-0.76434944	Non-Interacting
66	S	-1.1776457	Non-Interacting	66	S	-1.1687718	Non-Interacting
67	S	-0.96767276	Non-Interacting	67	S	-0.9390548	Non-Interacting
68	F	-1.0349774	Non-Interacting	68	F	-0.98125493	Non-Interacting
69	S	-1.0519283	Non-Interacting	69	S	-1.0413242	Non-Interacting
70	Q	-1.0165043	Non-Interacting	70	Q	-1.0056189	Non-Interacting
71	P	-1.0301084	Non-Interacting	71	P	-1.0140502	Non-Interacting
72	A	-0.95427477	Non-Interacting	72	A	-0.9495638	Non-Interacting
73	T	-1.0784161	Non-Interacting	73	T	-1.0786419	Non-Interacting
74	S	-0.95254672	Non-Interacting	74	S	-0.92545033	Non-Interacting
75	T	-0.9077265	Non-Interacting	75	T	-0.9022996	Non-Interacting
76	S	-0.56774113	Non-Interacting	76	S	-0.58594226	Non-Interacting
77	T	-0.62858207	Non-Interacting	77	T	-0.63028449	Non-Interacting
78	G	-0.47670062	Non-Interacting	78	G	-0.48366087	Non-Interacting
79	F	-0.08589069	Interacting	79	F	-0.10186104	Interacting

80	G	-0.50735971	Non-Interacting	80	G	-0.50449518	Non-Interacting
81	F	0.12438119	Interacting	81	F	0.1050222	Interacting
82	G	-0.38888995	Non-Interacting	82	G	-0.39747944	Non-Interacting
83	T	0.075502316	Interacting	83	T	0.041347035	Interacting
84	S	-0.17303079	Interacting	84	S	-0.19705206	Interacting
85	T	0.26220522	Interacting	85	T	0.21738126	Interacting
86	G	-0.03439857	Interacting	86	G	-0.06533779	Interacting
87	T	0.041335968	Interacting	87	T	-0.00559414	Interacting
88	A	-0.23688733	Non-Interacting	88	A	-0.29386109	Non-Interacting
89	N	0.05820687	Interacting	89	N	-0.01796369	Interacting
90	T	-0.24636999	Non-Interacting	90	T	-0.29849051	Non-Interacting
91	L	-0.41515678	Non-Interacting	91	L	-0.44493595	Non-Interacting
92	F	-0.75630273	Non-Interacting	92	F	-0.78610236	Non-Interacting
93	G	-0.51913145	Non-Interacting	93	G	-0.57522225	Non-Interacting
94	T	-0.57324359	Non-Interacting	94	T	-0.58675231	Non-Interacting
95	A	-0.90791421	Non-Interacting	95	A	-0.92717602	Non-Interacting
96	S	-0.56579222	Non-Interacting	96	S	-0.585956	Non-Interacting
97	T	-0.35803433	Non-Interacting	97	T	-0.38600291	Non-Interacting
98	G	-0.14025052	Interacting	98	G	-0.18097175	Interacting
99	T	-0.22947986	Non-Interacting	99	T	-0.24555298	Non-Interacting
100	S	-0.25903639	Non-Interacting	100	S	-0.26939633	Non-Interacting
101	L	-0.72867269	Non-Interacting	101	L	-0.7337462	Non-Interacting
102	F	-1.5055101	Non-Interacting	102	F	-1.4852729	Non-Interacting
103	S	-0.95047824	Non-Interacting	103	S	-0.93565842	Non-Interacting
104	S	-1.1651547	Non-Interacting	104	S	-1.1503788	Non-Interacting
105	Q	-0.52539393	Non-Interacting	105	Q	-0.52596819	Non-Interacting
106	N	-0.99015616	Non-Interacting	106	N	-0.98089022	Non-Interacting
107	N	-0.95779524	Non-Interacting	107	N	-0.94850312	Non-Interacting
108	A	-0.37984574	Non-Interacting	108	A	-0.38414445	Non-Interacting
109	F	-0.83849862	Non-Interacting	109	F	-0.83273493	Non-Interacting

110	A	-1.5557231	Non-Interacting	110	A	-1.5318974	Non-Interacting
111	Q	-0.8784608	Non-Interacting	111	Q	-0.87109706	Non-Interacting
112	N	-0.46131501	Non-Interacting	112	N	-0.462866	Non-Interacting
113	K	0.20884518	Interacting	113	K	0.18904333	Interacting
114	P	-0.23128636	Non-Interacting	114	P	-0.24036783	Non-Interacting
115	T	-0.2096631	Non-Interacting	115	T	-0.21949332	Non-Interacting
116	G	-0.15327384	Interacting	116	G	-0.16578721	Interacting
117	F	0.31626244	Interacting	117	F	0.29118409	Interacting
118	G	0.32724456	Interacting	118	G	0.30207997	Interacting
119	N	0.61000023	Interacting	119	N	0.57703863	Interacting
120	F	0.21606952	Interacting	120	F	0.19342053	Interacting
121	G	0.49218171	Interacting	121	G	0.46270835	Interacting
122	T	-0.07417111	Interacting	122	T	-0.08789188	Interacting
123	S	-0.28993514	Non-Interacting	123	S	-0.29757753	Non-Interacting
124	T	-0.64924421	Non-Interacting	124	T	-0.64966924	Non-Interacting
125	S	0.015272848	Interacting	125	S	-0.00252083	Interacting
126	S	-0.1431344	Interacting	126	S	-0.15467015	Interacting
127	G	0.012886299	Interacting	127	G	-0.00571911	Interacting
128	G	0.14138924	Interacting	128	G	0.11896952	Interacting
129	L	-0.49934315	Non-Interacting	129	L	-0.50369941	Non-Interacting
130	F	-0.23959612	Non-Interacting	130	F	-0.24987891	Non-Interacting
131	G	-0.35825414	Non-Interacting	131	G	-0.36702305	Non-Interacting
132	T	-0.72797192	Non-Interacting	132	T	-0.72785549	Non-Interacting
133	T	-0.6959551	Non-Interacting	133	T	-0.69549842	Non-Interacting
134	N	-0.44710493	Non-Interacting	134	N	-0.476782	Non-Interacting
135	T	-0.20438086	Non-Interacting	135	T	-0.24391674	Non-Interacting
136	T	-0.25912753	Non-Interacting	136	T	-0.28821713	Non-Interacting
137	S	-0.34135313	Non-Interacting	137	S	-0.33713262	Non-Interacting
138	N	0.14155492	Interacting	138	N	0.091506915	Interacting
139	P	-0.14956362	Interacting	139	P	-0.18465374	Interacting
140	F	-0.29269783	Non-Interacting	140	F	-0.28638717	Non-Interacting
141	G	-0.82672954	Non-Interacting	141	G	-0.81942059	Non-Interacting

142	S	-0.38685073	Non-Interacting	142	S	-0.42436374	Non-Interacting
143	T	-0.79021587	Non-Interacting	143	T	-0.80983266	Non-Interacting
144	S	-0.55153777	Non-Interacting	144	S	-0.58193886	Non-Interacting
145	G	-0.39222561	Non-Interacting	145	G	-0.43481243	Non-Interacting
146	S	-0.62833599	Non-Interacting	146	S	-0.66485185	Non-Interacting
147	L	-0.47007565	Non-Interacting	147	L	-0.48549155	Non-Interacting
148	F	-0.85006116	Non-Interacting	148	F	-0.85578051	Non-Interacting
149	G	-0.82726123	Non-Interacting	149	G	-0.82407064	Non-Interacting
150	P	-0.65474048	Non-Interacting	150	P	-0.64809849	Non-Interacting
151	S	-0.85642496	Non-Interacting	151	S	-0.84881163	Non-Interacting
152	S	-0.50571339	Non-Interacting	152	S	-0.50765667	Non-Interacting
153	F	-0.45379119	Non-Interacting	153	F	-0.45673466	Non-Interacting
154	T	-0.40774018	Non-Interacting	154	T	-0.41028561	Non-Interacting
155	A	-0.6588126	Non-Interacting	155	A	-0.65610309	Non-Interacting
156	A	-0.6588126	Non-Interacting	156	A	-0.6588126	Non-Interacting
